# A Practical Treatise on Auscultation

**Published:** 1842-04

**Authors:** 


ART. IV.—*A Practical Treatise on Auscultation.* By M. BARTH, M.D., and H. ROGER, M.D. Translated [from the French], with Notes, by P. NEWBIGGING, M.D. *Edinburgh*, 1842.—Small 8vo, pp. 398.

WE regard the original treatise of which this is a translation as decidedly the best that has yet appeared on the subject of Auscultation. Its great defect to the practical student is its passing entirely over the subject of percussion. We hope, on a future occasion, to notice the work more in detail; our object at present is to point out to readers ignorant of the French language, that they have now an opportunity of studying the treatise of MM. Barth and Roger in English—we wish we could add, *in good English,* but this we certainly cannot do. Although the translator justly condemns, in his preface, "the practice of introducing French phraseology into English writing, which has been long prevalent," he is himself one of the greatest sinners in this way we have met with for a long time. There is scarcely a page in the book which could pass with an English scholar as a sample of his genuine mother-tongue; so frequent is the recurrence of words, phrases, and idioms characteristically French. We cannot say that, on this account, the translation is unfaithful or the language at all unintelligible; but we say that the work is thereby rendered unpleasant to the reader of any taste, and is far from reflecting the credit on the translator which a version at once faithful and elegant could not fail to do.

